# The sedentary (r)evolution: Have we lost our metabolic flexibility?

**DOI:** 10.12688/f1000research.12724.2

**Published:** 2018-02-02

**Authors:** Jens Freese, Rainer Johannes Klement, Begoña Ruiz-Núñez, Sebastian Schwarz, Helmut Lötzerich

**Affiliations:** 1 Institute of Outdoor Sports and Environmental Science, German Sports University Cologne, Cologne, 50933, Germany; 2 Department of Radiotherapy and Radiation Oncology, Leopoldina Hospital Schweinfurt, Schweinfurt, 97422, Germany; 3 Laboratory Medicine, University of Groningen, University Medical Center Groningen, Groningen, 9713, Netherlands; 4 University College Physiotherapy Thim van der Laan,, Landquart, 7302, Switzerland

**Keywords:** Western diseases, Evolution, Hunter-Gatherer Lifestyle, Sedentary Lifestyle, Metabolic flexibility

## Abstract

During the course of evolution, up until the agricultural revolution, environmental fluctuations forced the human species to develop a flexible metabolism in order to adapt its energy needs to various climate, seasonal and vegetation conditions. Metabolic flexibility safeguarded human survival independent of food availability. In modern times, humans switched their primal lifestyle towards a constant availability of energy-dense, yet often nutrient-deficient, foods, persistent psycho-emotional stressors and a lack of exercise. As a result, humans progressively gain metabolic disorders, such as the metabolic syndrome, type 2 diabetes, non-alcoholic fatty liver disease, certain types of cancer, cardiovascular disease and Alzheimer´s disease, wherever the sedentary lifestyle spreads in the world. For more than 2.5 million years, our capability to store fat for times of food shortage was an outstanding survival advantage. Nowadays, the same survival strategy in a completely altered surrounding is responsible for a constant accumulation of body fat. In this article, we argue that the metabolic disease epidemic is largely based on a deficit in metabolic flexibility. We hypothesize that the modern energetic inflexibility, typically displayed by symptoms of neuroglycopenia, can be reversed by re-cultivating suppressed metabolic programs, which became obsolete in an affluent environment, particularly the ability to easily switch to ketone body and fat oxidation. In a simplified model, the basic metabolic programs of humans’ primal hunter-gatherer lifestyle are opposed to the current sedentary lifestyle. Those metabolic programs, which are chronically neglected in modern surroundings, are identified and conclusions for the prevention of chronic metabolic diseases are drawn.

*Charles Darwin: “It is not the strongest of the species that survives, nor the most intelligent, but the one most responsive to change.”*

## Introduction

During the longest time of *Homo sapiens*’ existence, approximately 99.5% or 84.000 generations ^1– 3^, humans’ daily survival was shaped by adaptation to a widespread range of different food sources (biodiversity), abundant daily exercise frequently under fasting conditions (foraging behavior), as well as unpredictable food supply (intermitted fasting), depending on the daily foraging success. Consequently, in the course of evolution, environmental stress forced the human species to develop an extraordinary flexible metabolism with multiple programs to guarantee energy equilibrium. Lipolysis, proteolysis, gluconeogenesis, ketone bodies and muscle-derived lactate as alternative energy substrates for cerebral neurons, provide examples by which natural selection tailored humans to buffer fluctuations in energy supply ^4^.

Until the Agricultural revolution around 12.000-10.000 BC, when humans began to store food on a larger scale for periods of shortage, survival would have depended on the adaption of energy needs to various climate, seasonal and vegetation conditions. Metabolic flexibility, optimized in millions of years of adaptation to environmental stimuli, safeguarded human survival independent of food disposability ^5, 6^. Due to an overflow of energy in modern times, humans have inevitably modified many evolutionary-based behavior patterns. As an example, when the body is threatened by energy depletion, hunger normally activates foraging behavior as a prioritized drive in both wild animals and hunter-gatherers, in order to anticipate new food resources ^7, 8^. Nowadays, hunting and gathering need only a few steps into the kitchen or a short drive to the local food store, which implies substantial energy intake by no or less energy expenditure of the locomotor system ^9– 11^.

In an evolutionary context, humans switched their primal lifestyle towards a plethora of energy-dense foods, persistent psycho-emotional stressors and a dramatic lack of exercise in record time. As a result of this ultra-rapid metamorphosis, humans progressively present metabolic disorders, such as the metabolic syndrome, type 2 diabetes (T2D), non-alcoholic fatty liver disease, certain types of cancer, cardiovascular disease and Alzheimer´s disease, wherever the sedentary lifestyle spreads in the world ^12– 17^. For more than 2.5 million years, our metabolic adaptation to seasonal food availability was considered to be an outstanding survival advantage. Nowadays, the same survival strategy in a completely altered surrounding is responsible for a constant body fat accumulation for periods of food deficiency, which will, however, very likely not appear again.

**Figure 1.**
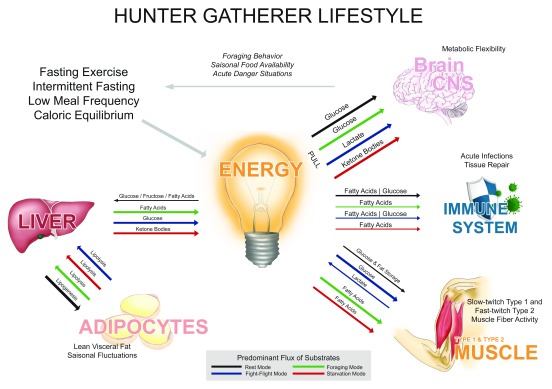
Metabolic flexibility in the hunter-gatherer lifestyle. The brain orchestrates its energy needs by either “pulling” energy from storage organs (from inside the body) in the daily foraging mode, in the short-term fight-flight mode or during long-term hunger in the starvation mode. If food is available, the brain is supplied with energy substrates through a “push” of nutrients (from outside the body) in the rest mode. If all programs are activated occasionally, the metabolic system holds the energetic equilibrium and body weight remains stable.

**Figure 2.**
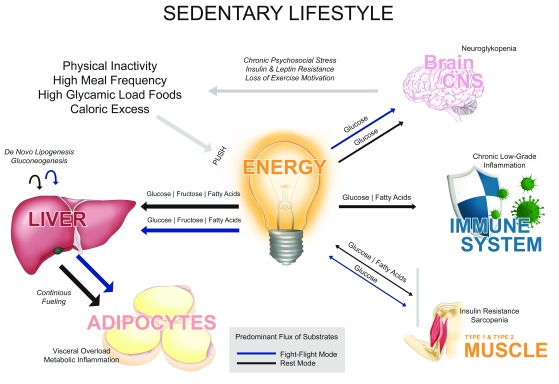
Metabolic inflexibility in the sedentary lifestyle. The “brain pull” (energy allocation from storage organs to the central nervous system) is compromised by “pushing” energy-rich nutrients into the metabolic system continuously. As a consequence, the foraging and starvation modes are both permanently unattended, which leads to a constant fueling of adipocytes, and in the long run to lipotoxicity, sarcopenia, low-grade inflammation and all associated metabolic diseases.

In this article, we argue that the strong increase of diseases related to metabolic abnormalities is largely based on a deficit in metabolic flexibility, a term initially stated by Kelley and Mandarino ^18^, which describes the way human physiology is adapted to alternate between lipid and carbohydrate fuels to cope with discontinuities of energy disposability in the environment. Here, we conceive this concept not only in a cellular but also rather systemic intention, and hypothesize that the modern energetic inflexibility, typically displayed by symptoms of neuroglycopenia, may be reversed by recultivation of suppressed metabolic programs, now obsolete in an affluent environment. In a model of predominant flux of energetic substrates between organs, the basic metabolic programs of humans’ primal hunter-gatherer lifestyle are opposed to the current sedentary lifestyle (see Figure 1 and Figure 2). In particular, we suggest that those metabolic programs relying on efficient fatty acid and ketone body oxidation are most of the time shut off in the modern lifestyle and have to be reintegrated in order to overcome the obesity epidemic – widely known as the breeding ground for most of the Western diseases (WD) ^19– 22^.

## Survival of the most flexible

### *Homo sapiens*: Adapted to scarcity rather than affluence

Evolution seems to have shaped the human metabolic system for ancient times of frequent energy deficiency rather than the present opulence, which entails adverse consequences, such as hyperglycemia, hyperinsulinemia, dyslipidemia and a chronic proinflammatory state induced by, among other things, accumulating visceral body fat ^21, 23– 26^. Hunger seems an adaptive response to food deprivation that involves neuroendocrine changes to motivate and enable food-seeking behavior ^27^. If evolution would not have developed a basic need of hunger-induced motivation for voluntary exercise in correspondence with energy allocation to the locomotor system, *Homo sapiens* would have become extinct ^7, 28^. Accordingly, if humans’ physical integrity would depend on a continuous ingestion of foods, humankind would not have been able to withstand climate changes, seasonal fluctuations, migration into different vegetation zones, physical conflicts with other hominoid groups, infectious diseases, and intoxications of rotten or dangerous food sources.

### It’s all about survival: Why evolution shaped metabolic flexibility

Anthropologic evidence suggests that, in the course of humanization, humans’ brain size increased at the expense of a shorter gut ^29, 30^. Presumably, humans found more energetically efficient and brain building nutrients ^31, 32^. In this regard, a long intestine became obsolete with the advantage to expend less energy on digestion, which allows energy distribution to a growing, energetically more demanding brain and the immune system ^30, 33, 34^. When ATP-levels of neurons decrease, the provision of energy to the brain enjoys a privilege, due to limited storing options in cerebral tissues. As a consequence, the metabolism must favor an ongoing energy supply to the brain in any kind of situation ^35– 37^. It is not by coincidence that glucose is the first macronutrient absorbed in the small intestine. Although hypothetical, this fact could underline the rarity of sugar in ancient times. At any rate, cerebral neurons usually favor glucose as their primary energy substrate. However, they are capable of either utilizing ketone bodies from visceral fat cells in starvation periods ^33, 38, 39^ or muscle cell-derived lactate in intense activity (see below). Ketone bodies are the major alternative fuel source for the brain, providing approximately one third of its energy requirements after only four days of very low carbohydrate intake ^40^. Ketone utilization is thereby increased in proportion to ketone body blood concentrations and inversely related to the brain’s glucose utilization. One mechanism seems to involve a downregulation of glycolysis in astrocytes, which sit between neurons and the vasculature, in this way securing a steady glucose supply to neurons ^41^. Interestingly, astroglia and neurons seem to be capable of producing ketone bodies by themselves under conditions of increased AMPK activation, such as hypoxia or hypoglycemia ^42^.

In this context, the selfish brain clinical research group (Lübeck, Germany) differentiates between ingestive and allocative behavior (see Figure 1 and Figure 2). In other words, if a human being’s brain is adapted to satisfy its energy demands by ingesting high-glycemic dense nutrients (push-principle), it forfeits the ability to allocate (pull-principle) substrates from peripheral storage organs, i.e. glucose and ketone bodies from the liver, free fatty acids from adipocytes and lactate from skeletal muscles ^33, 37, 38, 43, 44^. Instead, in affluent, sedentary conditions high-glycemic load foods are continuously pushed into the metabolic system (see Figure 2), promoting ongoing ingestive behavior, followed by insulin and leptin resistance in the long run. Constant food availability and physical rest accompanied by chronic psychosocial stress insidiously impairs allocative behavior, leading to overnutrition, caloric excess and a persistent fueling of adipocytes. As described, such metabolic inflexible individuals often perceive hypoglycemia (neuroglycopenia), answered by further food intake – the beginning of a vicious circle.

### The evolution of obesity: From thrifty genes to uricase mutation

Storing excessive calories as fat in affluent times is an existential meaningful adaptation in the context of food shortages. Based on this hypothesis, which has been discussed controversial in the field of anthropology, the geneticist James Neel postulated in 1962 his much-cited thrifty gene hypothesis, which states that genes have been positively selected for efficient intake and utilization of food − good for hunter-gatherers in a feast/famine environment, bad for modern people in a world of plenty. In his field studies on Yanomami Indians of the Amazon region, Neel observed that individuals were completely free of overweight and T2D ^45– 47^. In contrast, the incidence of T2D in Western industrial countries is at an alarming status ^15, 48^. Obviously, people currently face a dramatic mismatch between their genetic heritage and today's sedentary lifestyle.

Proponents of Neel´s approach consider recurring famines as the driving force for the natural selection of thrifty genes ^9, 49, 50^. Neel proposed that muscles become insulin resistant in a metabolic starvation program to maintain high glucose levels for the central nervous system, while lipolysis is upregulated to provide fatty acids for muscles and ketone bodies for the brain. Speakman challenged this hypothesis later on ^51^ with the counter-argument that if overweight would have been so beneficial in the past, there wouldn’t exist so many slim people nowadays. He opposed the so-called predation release hypothesis and blamed the loss of natural predators for the obesity epidemic, which was forced back around two million years ago by the development of social behavior, weapons, and fire ^47, 51^. Furthermore, Berbesque *et al*. ^52^ found no evidence for assuming that Paleolithic hunter-gatherers would have frequently encountered serious famine conditions, and argued that a cultural or genetic adaption to strive for the most energy-dense foods and to consume them as quick as possible rather than storing them could be a better alternative to the thrift gene hypothesis for explaining the adverse consequences of today’s affluent environments.

However, the hypothesis of Neel gained new support through the discovery of a mutation in uricase which occurred 13–17 million years ago, when human ancestors were still great apes and living conditions became colder ^53^. Uricase degrades uric acid, which is produced by the breakdown of DNA substances from food or the body's own cells ^54^. Uric acid in turn facilitates conversion of fructose into fat stores. Thus, as winter periods became dry and cool in the middle Miocene (approximately 15 million years ago) Hominoids would have become able to better metabolize fructose into fat. However, a survival advantage in the past has now become a curse. Since the invention of the enzymatic extraction of fructose from corn in the 1980s, refined fructose is attached to most industrial food products, especially sugar-sweetened beverages ^55, 56^. Contrary to Paleolithic times, no winter or dry period provides a degreasing any longer. Apparently, the human metabolic equilibrium seems to be literally depending on differences in food availability, which implies an alternating depletion and reload of energy storage. Anyhow, given the observation that obese individuals show much higher levels of uric acid as slim fit people ^57^ the uricase mutation can be seen as a confirmation of Neel´s thrifty gene hypothesis.

Regardless of the genetic debate, obesity establishes where industrialization arrives ^58^. In this context, a clear trend can be observed in developing countries. If people leave their rural habitat and move to the cities, obesity and T2D are on the fast lane ^13, 17, 59^. Modern inventions to make people’s physical life more convenient are extremely successful (pedelecs, ready-to-eat-food, home automation etc.). When modern humans obtain the opportunity to save energy, they unconsciously select convenience, e.g. parking close to the entrance of a shopping center to avoid walking distances, favoring packaged food instead of spending hours preparing meals. Undoubtedly, this energy-saving behavior must have been shaped by evolution based on fluctuations in energy availability. The screw of time cannot be turned back, but the crucial question is: How can we remain metabolically healthy in a digital and saturated environment, which promotes sedentary behavior?

## The hunter and gatherer lifestyle

### Morning has broken: The foraging mode

Foraging is the vital need to search for food based on locomotion, which requires energy expenditure in the form of running, sneaking or sprinting to plunder big or small game or climbing and stooping in order to collect honey, berries, nuts, seeds and plants. Admittedly, human beings’ ancestors always had to balance their investment of stored energy against the assumed outcome of a supposed foraging success ^60^ to get physically active. The light-dark cycle forced organisms to develop a circadian rhythm to save energy and avoid predation by specialization to an active (light) and inactive (dark) period ^61^. Independent from hunger, thirst or other motivational drives, the circadian rhythm ensures the initiation of the so-called cortisol awakening response ^62^. One major task of the glucocorticoid cortisol is binding to fat cells and glycogen storing tissues in the liver and skeletal muscles, when blood glucose concentration is low ^63^. Cortisol levels increase autonomously in the morning before awakening to provide preferentially glucose for the brain and fatty acids for skeletal muscles to support foraging behavior, or physical activity in general. All behavior patterns in the context of physical activity are associated with energy demands of neurons and skeletal muscles, although energy intake was never predictable before the era of agriculture. As a result, to maintain homeostatic levels in terms of different environmental stimuli, humans inevitably became highly flexible in energy allocation ^64^. In the morning, the circulating amount of glucose rises, a fact known as the dawn-phenomenon ^65^. Simultaneously, the non-insulin-dependent glucose utilization is increased in skeletal muscles. Even muscles at rest demand more glucose in the morning than in the afternoon ^66^. Animal studies show that if the metabolic rhythm is disturbed, e.g. due to night shifts or by exchanging the physical activity phase with the rest phase, the risk of glucose intolerance and obesity increases ^67^. In contrast, fasting during the activity phase prevents metabolic disorders ^68^. Those insights illustrate that autonomic endocrine functions prepare human organisms in the morning for physical activity independent of energy intake.

Across cultures and epochs, people always tend to consume three meals a day ^69^. At daytime, leptin levels decrease and hunger signals emerge to increase sensitivity to brain reward systems, which boost the motivational drive of physical activity, facilitating foraging behavior ^70– 72^. Furthermore, lipolysis is upregulated due to activation of the hypothalamic-pituitary-adrenal (HPA) axis, the central nervous system and glucagon from pancreatic islets to induce ß-oxidation of fatty acids, breakdown of glycogen and gluconeogenesis. In contrast, during night time, leptin levels increase, since appetite needs to be reduced in order not to disturb sleep by hunger signals. In addition, due to the fact that the activity of the HPA axis and the suprachiasmatic nucleus (SNS) are low, ketone body utilization provides energy for the brain, while growth hormone stimulated gluconeogenesis allocates glucose to the immune system ^73– 76^.

Responsible for controlling the circadian rhythm is the SNS. This core area of the hypothalamus can activate or silence various organs via the central nervous system. Both, the sympathetic and parasympathetic part of the central nervous system can subtly distinguish between intra-abdominal or subcutaneous fat by activating storage types depending on the vital necessity of the body ^61, 69^. The vastest release of adiponectin, produced by adipocytes as a fundamental signal for the brain to modulate food intake ^77^, occurs at 10 a.m. in the morning, synonymous with the greatest glucose tolerance ^78^. Taken together, in the morning, human organisms are metabolically well prepared for physical activity, independent of energy intake. Accordingly, it seems contradicting that leading authorities throughout the developed world recommend high-glycemic foods for breakfast in order to provide energy for a sedentary daily routine, since cortisol has been generating energy at sunrise for more than 2.5 million years without energy intake ^62, 79, 80^.

### Old danger signals: The fight-flight mode

As all living organisms, humans maintain a dynamic homeostasis, which is constantly challenged by internal and external stressors ^81^. The fight-flight response represents the archaic physiologic response to a threat from a danger signal ^82, 83^. In the history of mankind, humans always faced external threats, such as predators, storms, fire, hostile peers, and toxins in plants, meat or water, as well as internal immunological hazards, such as viruses, bacteria or fungi ^84^. Dangerous situations were usually solved within minutes/hours/days (e.g. escape from predators) or at least weeks, because fighting acute infections or healing trauma could only last until all energy stores were empty, which means approximately 19 to 43 days for females and 28 to 41 days for males ^6, 85^.

In general, fighting and fleeing require augmented energetic demand to support locomotor behavior. Energy allocation is a prioritized issue in this context. If rapid muscle activation is important for survival, the SNS allocates energy to the brain, heart and large skeletal muscles ^83, 86^. Blood flow in muscles can increase from 1200 to 22,000 ml/min. In contrast, blood flow to organs such as the kidneys or the viscera, which are energetically neglected in acute danger situations, decreases significantly ^87^. The interplay of SNS-derived catecholamines and the secretion of cortisol, delivered by the HPA-axis, mobilizes glycogen and fatty acids from storage organs (muscles, liver, fat tissue) to provide fuel for the locomotor system. The reversion of an upregulated stress system to homeostasis is metabolically demanding. In case of an acute infection, each degree above normal body temperature costs a daily metabolic increase of 7–13% ^88– 90^. Already moderate infections boost gluconeogenesis up to 150–200% and severe infections cost 15–30% of body weight due to a persistent elevated basal metabolic rate of 25–55% ^91, 92^. Consequently, based on volatile environmental conditions, it becomes clear that humans’ Paleolithic ancestors could not afford a chronically activated immune system, because hypermetabolism during an acute inflammation would become life-threatening if energy stores run empty. However, modern danger signals (e.g. psycho-emotional stress, high meal frequency, physical inactivity) chronically affect humans’ immune system ^93– 95^. This persistent low-grade inflammation state is displayed by the mounting epidemic of non-communicable diseases in Western societies ^96– 98^.

In acute danger situations, type 2 muscle fibers demand high amounts of glucose to initiate fighting or fleeing behavior ^18^. In order to avoid a glucose conflict between the brain and contracting type 2 muscle fibers, evolution created alternative pathways to secure the energy demand of the brain. During high-intensity work, type 2 muscle fiber derived lactate is increasingly adopted and oxidized by type 1 muscle fibers and organs such as the heart and brain ^99^ or – to a lesser extent of up to 20% clearance – recycled to glucose in the hepatic Cori cycle ^100, 101^. Similar to ketone bodies, brain lactate uptake is accomplished via monocarboxylate transporters, proportional to the arterial concentration of lactate, and able to reach a similar rate as glucose uptake, yielding up to one third of the carbon sources for oxidative phosphorylation ^102^. Frequent exercise improves lactate removal ^100^ which subsequently may stimulate the production of brain-derived neurotrophic factor and improve cognitive performance ^103^.

The flexibility to switch between metabolic pathways within seconds, prevented metabolic impairments of people’s physical and cognitive performance, which was vital for survival in a hostile environment ^5, 104^. Danger and stress factors, affecting the Paleolithic ancestors, were acute within a relatively short timeframe and induced physical activity (escape) or sickness behavior and anorexia in case of an acute inflammation to overcome major wounds or severe infections as soon as possible ^105^. Certainly, nowadays, modern humans suffer from chronic mental stress, which can last for days, weeks or even years with severe metabolic and psychiatric consequences ^84, 106, 107^.

### Hungry eyes: The starvation mode

Unlike today, human beings’ Paleolithic ancestors could not predict meals. Human nutrition used to be subject to weekly, monthly as well as seasonal fluctuations ^108– 110^. Anyhow, in periods of fasting, humans should have been able to use stored energy, if necessary, for many hours or even days without any food supply. In other words, if pushing exogenous energy into the body used to be impossible due to food shortage in the past, energy allocation from energy stores to target tissue seems the only alternative to survive periods of food insecurity. For that reason, in late summer or at the end of a rainy season (the time of plenty), it became extremely important for our Paleolithic ancestors to take advantage of easy digestible carbohydrates, such as ripe fruits, to build up body fat, preparing for a scarcer food supply during winter or dry season (the time of caloric or carbohydrate restriction) ^111^. *Homo sapiens* adapted to these seasonal changes by developing several survival mechanisms. One example is given by the fructose transporter, which enables fruit-derived fructose to be allocated into cells independent of insulin ^112^. In contrast to glucose (20%), nearly 100% of all ingested fructose is immediately converted into fatty acids and then further esterified to storage triacylglycerols by the liver ^57, 113– 115^. Adipose tissue, responsible for 60% of all triacylglycerol accumulation, can store large amounts of excess free fatty acids. The mechanism to convert fructose into fat was an important survival advantage for our ancestors, particularly if facing seasonal starvation periods. In the animal kingdom, hibernating species were not the only species that developed mechanisms to store energy, and protect themselves in times of food shortage. For example, northern elephant seals can fast for 60 days ^116^ and king penguins survive 5 months without energy intake ^27^. Adipose tissue of modern humans contains approximately 13 kg fat or 500,000 kJ, which would theoretically last for 2.4 months ^34^. As a result, survival was not only dependent on fitness, as argued by Charles Darwin ^117^, but also on fatness, since overweight can guarantee weeks of survival without any food supply ^118^. Alternatively, it can be imagined that early humans had to endure times not necessarily of reduced energy intake, but drastically reduced carbohydrate intake, if animal, but not plant foods were abundant. Physiologically, this situation is similar to fasting because it is mainly (exogenous) glucose that interferes with the adaptive response to fasting, while fat is a neutral macronutrient in this respect ^119^. The nuclear peroxisome proliferator activating receptors that stimulate fat oxidation and ketogenesis are activated by free fatty acids, which can stem from both exogenous, preferably obtained from hunted animals, or endogenous fat, released during times of food scarcity ^120^. It is interesting that across cultures, indigenous humans share a high preference for animal fat ^121, 122^, underscoring the importance of this macronutrient for human health and survival in times when only animal food was available.

Thus, intermittent fasting and longer-term caloric as well as carbohydrate restriction are parts of our genetic heritage. All three can lower glucose, insulin, triglycerides and lipid accumulation and, at the same time, increase insulin sensitivity, while increasing HDL-cholesterol ^123^. Intermittent fasting itself was proven to significantly reduce low-grade-inflammation by degrading IL-6, TNF-alpha and CRP ^109, 124, 125^. Studies with mice also demonstrated that time-restricted feeding without reducing caloric intake can prevent metabolic diseases, if fed a fattening diet ^68^. Other evidence reveals that intermittent fasting and caloric restriction both enhance cardiovascular function and increase insulin sensitivity ^118, 126^. In humans, glucose homoeostasis in fasting periods are initially bypassed by liver glycogen (0.5 days) and proteolysis mainly from muscles (from day 1 to 3). From day 3 onwards, liver-derived ketone bodies are used as a glucose substitute for the brain, muscles and immune cells ^74^. Recapitulating, nature provides seasonal fluctuations of food availability, leading to alternating periods of fatness and leanness, whereas today, energy abundance determines modern life throughout the year.

### Work first, then the reward: The rest mode

As mentioned before, food seeking behavior is an evolutionary-based motivational drive to forage, in order to maintain energy balance on homeostatic level. Foraging success in the form of food represents the primal reward for a physical effort, which activates parasympathetic circuits to promote digestion, recovery of energy stores and repair ^105, 127– 129^. In order to avoid overnutrition, our metabolic homeostatic system involves several hormonal regulators of hunger and satiety, such as leptin, ghrelin, and insulin, which act on hypothalamic and brainstem circuits to inhibit further feeding. Dysfunction of this homeostatic system can result in a persistent state of positive energy balance, overeating and obesity. Palatable high caloric foods might be a major reason for this dysfunction in the Western world by exchanging the regulation of food intake from homeostatic to rewarding pathways ^106^, since food intake displays much more than balancing energy status. Humans consume food also for its hedonic properties independent of energy status, revealing that the brain reward system plays an important role in feeding behavior ^130^. A pivotal component of reward and motivation circuitries is cerebral neurons, which originate in the ventral tegmental area. These cells send projections to the nucleus accumbens, a structure deep beneath the frontal cortex, by utilizing the essential neurotransmitter dopamine. Termed as the mesolimbic dopamine system, this brain region is responsible for the processing of aversion, motivation, pleasure and reward, and in consequence is linked to encoding rewarding stimuli, such as comfort food, sex, sports (runner´s high), and addictive substances (caffeine, ethanol, nicotine, heroin, etc.) ^127, 129, 131^. One hypothesis for abnormal hedonic behavior displayed by overconsumption of high-glycemic carbohydrates could be that modern processed foods lack in essential amino acids. In addition, processed foods contain less micronutrients compared to the Paleolithic food selection ^132, 133^. A poor nourishment status in both essential macro- and micronutrients might be at least co-responsible for reward deficiencies, which result in ongoing food seeking behavior rather than resting after enjoying the foraging success.

Affluence of energy-dense food is considered a major environmental risk factor for obesity ^134^. Rats with extended access to palatable food rapidly gain weight and reward deficits ^127^. Leptin resistance could be the functional link between reward deficiency and persistent ingestive behavior. Leptin receptors are expressed on dopamine neurons in the ventral tegmental area ^135^. As proven in rodents, leptin infusions into the ventral tegmental area inhibit the activity of dopamine neurons and decrease food intake ^136^. Further, knockdown of leptin receptors in the ventral tegmental area enhance food intake and preference for palatable food ^136^. Administered from adipocytes leptin acts as an inhibitor of mesolimbic dopamine transmission in physiological conditions to interrupt food seeking behavior if storage organs are loaded. Obese individuals demonstrate increased activation of reward circuitries in response to palatable food compared with lean controls ^137, 138^. Apparently, hypersensitivity of reward circuitries predisposes to overnutrition and weight gain ^139^. However, during the further weight gain process, rewarding effects are blunted to reach hyposensitivity, which perpetuates overnutrition in order to overcome reward deficits ^140, 141^. In conclusion, effort and reward are directly interconnected to silence dopamine-driven behavior. In Paleolithic times, reward substances were limited, whereas today modern humans are surrounded by a plethora of rewarding stimuli in a nearby environment, which can be addictive and finally inhibit physical activity.

## The sedentary lifestyle

### Modern habitat: A life in mental stress and physical rest

The current rise in metabolic diseases is descriptively explainable with a neglect of the important ancient metabolic programs. Our metabolic flexibility qualified us to be the most dominant of all living species on Earth. However, our energetic flexibility fell victim to major technical achievements of the last 150 years, a blink of an eye in an evolutionary point of view. For clarification, since 1887 food is stored in refrigerators, since 1900 muscle-derived energy expenditure is saved by the use of cars, and since 2001 people meet friends all around the world via the world-wide-web without investing a single calorie in locomotion. The inventions of the last three centuries increased physical inactivity. Moreover, the launch of high fructose corn syrup (HFCS)-production in the late 1980s has set up the crown on a sedentary lifestyle with abundance of nearby and easy to digest energy. Ever since, the obesity epidemic proceeds relentless ^4, 114, 142, 143^.

Many modern humans capitulate from the speed of innovation. Stress-related disorders are increasingly exorbitant ^144, 145^. Chronic psycho-emotional stress factors, such as liabilities, job loss or time pressure, face an archaic stress system (fight-flight-mode), made to overcome acute danger situations rather than long-term stress. Locomotion, the evolutionary designed solution to dismantle acute stress reactions, is no longer an option in a sedentary working ambiance. As a result, sedentary lifestyle in association with chronic psycho-emotional stress and a plethora of high-glycemic load foods, lead to a constant fueling of adipocytes, organs and other tissues ^146^ (see Figure 2). Accordingly, we blame here three essential lifestyle factors for obesity and its related diseases:

1) Chronic high-glycemic food availability in the nearby environment

2) Loss of existential motivation for physical activity

3) Increase of psycho-emotional stress not ventilated by muscle activity

### Good calories, bad calories: The burden of chronic energy availability

The modern lifestyle promotes continuous fueling of adipocytes (see Figure 2). In response to adipose hypertrophy, neutrophils, macrophages, natural killer cells and other immune cells infiltrate adipose tissue and in the further process develop an inflammatory environment, based on a phenotypic switch from anti-inflammatory M2- to pro-inflammatory M1-macrophages ^78, 147^. This condition induces a systemic low-grade inflammation, characterized by elevated levels of TNF-alpha and IL-6, which contributes to and amplifies insulin resistance ^19, 21, 23, 148, 149^. As accumulation of adipose tissue approaches its limit, the body is compelled to use organs and other tissues for fat storage, such as skeletal muscles, pancreas, and liver, and as a second choice the heart, kidneys and bones, thereby boosting insulin resistance ^23, 150– 152^. As a matter of fact, the liver is one of the organs affected first. A non-alcoholic fatty liver (NAFL) is present in nearly 25–50% of the general population in Western countries ^152, 153^ and strongly related to T2D, hyperglycemia, hyperinsulinemia, low HDL-cholesterol and represents an independent risk factor for cardiovascular diseases ^154^. It is striking that changes in lifestyle patterns opposing those of a sedentary lifestyle such as physical activity, exercise, periods of calorie restriction or cold water immersion seem to exert holistic beneficial effects on body composition, NAFL and low-grade inflammation ^155, 156– 160^.

Glucose is the primary nutrient for neurons and red blood cells, but rather rare in our primal habitat. Therefore, honey has always been a privileged food for hunter-gatherers, and accounts for a substantial proportion of kilocalories in some primal living societies, since honey is the most energy dense food in nature. In the Hadza diet (a hunter and gatherer tribe in Tanzania), honey contributes roughly 15% of their total energy intake ^161^. At least in warm areas, honey might have been a regular part of humans’ hunter-gatherer past, since current indigenous people take not inconsiderable risks to gain access. All of humans closest relatives, like chimpanzees, bonobos, gorillas and orangutans, consume honey. Honey is the sugar source par excellence, as its dry matter contains 95% of carbohydrates, averaged 40% fructose and 30% glucose ^162^. For this reason, honey is the ideal fuel to supply the central nervous system with glucose and simultaneously refill fat stores with fructose for both animals and humans, living in the wild to stand seasonal fluctuations of energy availability. Today, fructose is ubiquitous as a low-cost sweetener. In the late 1980s, the US food industry began incorporating HFCS into soft drinks and industrial foods, greatly promoting the persistent caloric surplus in present day societies ^163^. It has now become clear that regular consumption of processed and high-glycemic load foods, especially sugar-sweetened soft drinks ^55, 57, 164^ , supported by a growing lack of exercise, change human body composition towards an accumulation of visceral fat mass and sarcopenia ^165, 166^. As a result, the reduction of sugar-sweetened foods might be one of the major key factors to overcome the obesity epidemic.

### Sit and wait: Metabolic consequences of sedentary behavior

The capability to store fat inside muscles could have been an evolutionary advantage to preserve the existential motivation to hunt and gather food in times of shortage. Observational studies of recent hunter-gatherer societies, such as the Hadza in Tanzania, forager groups of the Andaman Islands or the San people in the Kalahari Desert, reveal that men usually walk long distances for hunting animals, while women search for collectable food and fresh water in the nearby environment ^60, 167– 169^. Most physical activities of the mentioned aboriginal tribes are movements at low intensities, where skeletal muscles use fatty acids as the primary energy source. Oxidative type 1 muscle fibers are characterized by a higher fat content than glycolytic type 2 fibers. They contain more mitochondria, which utilize stored intramuscular fat as a rapidly available energy source. Therefore, oxidative slow-twitch muscle fibers are perfectly designed to overcome long distances in order to forage or see new biospheres. Interestingly, high intramuscular fat is associated with both the development of insulin resistance and the effects of endurance training, known as the training paradox in sports physiology ^170^. Although fat accumulation in muscles of obese people is a consequence of a spillover of nutrient excess in sedentary terms, it has been suggested that the supercompensation of intramuscular fat after fasting periods or exhaustive workouts could have been beneficial in human evolution to permit physical activity in times of food shortage ^171^. *De facto*, if prolonged exercise (hunting big game or discovering new hunting grounds) was vital for survival in the past, high intramuscular fat stores secure ß-oxidation in active muscle cells ^18, 104^. In this situation, glucose can be spared for the central nervous system (at daytime), glucose-depending cells (e.g. red blood and kidney cells) and the immune system (at night) – an excellent metabolic division of work on the scarcity of glucose to guarantee a constant glucose influx to glucose-depending cells ^34, 172, 173^.

Nowadays, the biggest metabolic challenge for obese, (pre-)diabetic, metabolically inflexible healthy individuals, but also athletes on high carbohydrate diets, is to maintain blood sugar levels in an affluent environment. In conditions of high locomotor speed, chronic mental activation or insulin resistance, there is an increasing demand of glucose either by type 2 muscle fibers (in high-intensity conditions) or the brain (in chronic mental activation or under insulin resistance). In dangerous situations, when muscle activity beyond 70% VO2max is needed for survival (or nowadays in competitive sports), the utilization rate of muscle and liver glycogen can come to its limits ^33, 104, 172^. Consequently, the euglycemic state is compromised and metabolically inflexible athletes typically show symptoms of neuroglycopenia, which are commonly answered or prevented by the intake of carbohydrate-rich foods. This situation would be different in “keto-adapted” athletes, who are able to sustain fat oxidation at high intensities and display high levels of ketone bodies during exercise, which can be used by the brain. These capabilities have been impressively documented by Volek *et al.* ^173^ , showing that in keto-adapted endurance athletes peak fat oxidation is shifted towards higher intensities (70 ± 6% of VO _2_max) and displays absolute values more than twice as large (1.2 ± 0.2 g/min) as those of endurance athletes on a high carbohydrate diet. Apart from athletes, owing to the digital sedentary lifestyle that affects all aspects of life, modern humans frequently walk less than 1 km per day, their physical activity level has fallen below 1.7, and their VO _2_max fitness markers are of alarming status ^1– 3, 10, 174, 175^. In such conditions of physical inactivity, the need for carbohydrates as a fuel is at a minimum level. By contrast, most authorities in the Western world recommend at least 50% of the daily caloric intake as carbohydrates for a sedentary lifestyle ^176– 179^. Although the brain accounts for about 2/3 of the total daily glucose turnover ^180^, the brain is not capable of accumulating large amounts of glucose. Since high blood sugar levels are pro-inflammatory ^9, 181, 182^, excess glucose, if not utilized by the brain or muscles, has to be metabolized in the liver as fat, continuously fueling adipocytes (see Figure 2)

Even humans with a constitutional high percentage of fast-twitch fibers, which are competent to burn high amounts of glucose within a short timeframe, have given up this opportunity to clear glucose from the blood in a digital world. These subjects rapidly store fat given a Western diet in physical rest ^104, 183– 186^. In Paleolithic times, this adaptation could have been a survival advantage in specific environmental conditions, but, nowadays, it seriously impairs metabolic health.

### In a hurry: Modern stressors and visceral fat storage

Like all biological systems, physical and mental stress is mutually dependent on the dynamic homeostasis. For example, it is well established that an overload of exercise activates humans’ immune system and provides a higher vulnerability to infectious diseases ^187– 190^. In modern societies, physical workload is increasingly detached from intellectual workplaces. In the light of working conditions at present, a significant trend can be observed that once humans enter sedentary professions, they become overweight ^191^. Nowadays, in an affluent environment under the influence of multiple new stressors, people’s primal systems can come to their limits. Social hierarchy is a perfect example to display individual stress load. Sapolsky ^192, 193^ found that in baboons social subordinance and social isolation is associated with manifestations of hypercortisolism. In modern working organizations, the mean cortisol level is higher in individuals with low socioeconomic status because of high job demands. The same holds true for low job control in general ^194^. Many overstressed people experience long-term stress since they are not capable of changing their self-chosen or enforced environment ^195^. From a biological perspective, intense muscle activity is the natural response to stressful or dangerous stimuli. At the present day, exercise has lost its primal role due to the fact that modern humans living in a digital environment do not secure their subsistence by physical workload anymore. Exercise no longer serves as either foraging behavior or escaping from dangerous conditions and fighting predators. If modern humans are mentally hyperactive (e.g. chess playing on world class level), skeletal muscles become insulin resistant for a short time, to redirect glucose towards the central nervous system ^196^. In this case, humans’ brains rapidly demand glucose replenishment when stressors continue ^37, 89, 197^. In the long run, a temporary functional insulin resistance, which secures survival of neurons in acute danger or infectious situations, passes over into a persistent insulin resistant state. This condition was recently viewed as protective to avoid abundant intracellular substrate accumulation, especially in liver and muscle cells ^198^. However, as a consequence of prolonged insulin resistance, insulin receptors are blunted and transmembrane signaling is attenuated ^199– 201^.

## Conclusions

A return to Stone Age conditions is neither possible nor desirable. Plants are highly cultured, movement has lost its existential necessity to forage, and stress influences act no longer as acute danger signals but are subtly persistent. Paleolithic times were certainly no paradise. Modern achievements such as food preservation, mechanization, antibiotics, high-tech medicine and the virtual elimination of infant mortality have significantly increased life expectancy in the Western biosphere ^202^. Even contemporary hunter-gatherers do not reach the age of modern humans, predominantly due to much higher rates of infant mortality and serious, unhandled infections ^203^. Nevertheless, the era in which modern humans suffer from chronic diseases is extending ^204^ and metabolic disorders occur much earlier ^16, 21, 205, 206^. Periodic fat gain is considered physiologic, whereas persistent overweight contradicts humans’ evolutionary designed metabolic flexibility and promotes a chronic low-grade inflammation, which can be considered as the hotbed for WD. Apparently, since evolution is a slow-acting process, modern humans are not yet well adapted to a sedentary lifestyle. Approximately 99.5% of our existence, we lived as hunter-gatherers ^1, 2^, being metabolically prepared for any kind of environmental condition. According to the present article, today´s sedentary lifestyle limits our primal metabolic flexibility to a stress and rest mode (see Figure 1 and Figure 2), while the starvation and foraging mode are consistently neglected if not consciously integrated in daily life. Regular activation of these modes, however, appears necessary to maintain metabolic flexibility in a modern habitat, characterized by energy abundance, prolonged psychosocial stress and physical inactivity. While certain drugs or “calorie restriction mimetics” such as metformin or resveratrol appear as a convenient way of activating signaling networks associated with the starvation and foraging mode, they are not able to fully mimic the complex adaptions and molecular signaling taking place with actual lifestyle interventions. For the latter we argue that no specific diet, exercise or anti-stress program must be followed if behavior is adjusted based on human being’s evolutionary heritage. Examples include, but are not limited to, periodic fasting or calorie restriction (e.g., once a year as traditionally practiced within the world religions), occasional meal skipping, ketogenic diets (e.g., during the winter months) and a combination of endurance and strength training several times per week.
