# Supplementary material for: The sedentary (r)evolution: Have we lost our metabolic flexibility?
Source: F1000Res. 2018 Feb 2;6:1787. Originally published 2017 Oct 2. [Version 2] doi: 10.12688/f1000research.12724.2 (PMC5710317; doi:10.12688/f1000research.12724.2)
Supplement: Peer review history

# Peer review: Referee response for version 1

**DOI:** 10.5256/f1000research.13782.r28692

**Published:** 2017-12-06

**Authors:** Shuzo Kumagai

**Affiliations:** 1 Faculty of Arts and Science, Kyushu University, Kasuga City, Japan

This article published by Jens Freese et al. discussed the potential explanations of metabolic epidemic from the viewpoint of the metabolic flexibility sharped by evolutionary through millions of years, and a combination of present-day continuous food abundance, sedentary lifestyle, and psycho-emotional stress. In their manuscript, Jens Freese et al suggested metabolic programs relying on fatty acid and ketone body oxidation are most of the time shut off due to modern lifestyle and have to be reintegrated in order to overcome the obesity epidemic.

 The storyline of ‘Survival the most flexible’ section could be more clear if the description could be more concise and focusing on how evolution sharped the metabolic flexibility. For example, the content of ‘It’s all about survival: Why evolution shaped metabolic flexibility’ seems discussed the interplay of metabolic programs in brain and other organs (e.g., skeletal muscle and adipocytes) which could be combined into the first section. The sections regarding uricase mutation could also be more straightforward by describing the importance of this mutation (e.g., more efficient metabolism of fructose and fat stores) in the way of evolution.  

 In the section of ‘Sedentary lifestyle’, the authors described a modern lifestyle which is opposite to the ‘The hunter and gatherer lifestyle’. Accordingly, this section could also benefit from a more concise storyline by comparisons such as sedentary behavior vs foraging mode.  Besides molecular-level evidence, there are also quite a lot of epidemiological evidence has highlighted the importance of environmental, and behavioral (dietary and sedentary behaviors) factors in the progress of metabolic health outcomes. Incorporation of these evidences will provide more insight into how to re-cultivate of suppressed evolutionarily metabolic programs and make the proposed model more convincing and readily understood for the researchers in the field as well as the general readers.

I have read this submission. I believe that I have an appropriate level of expertise to confirm that it is of an acceptable scientific standard, however I have significant reservations, as outlined above.

# Response

**Published:** 2018-01-29

**Authors:** Jens Freese

**Affiliations:** Deutsche Sporthochschule Köln, Germany

**We would like to thank all reviewers for their constructive comments and positive feedback to our article. We have addressed their comments as described below, and think that they greatly helped to improve our article further.**

 The storyline of ‘Survival the most flexible’ section could be more clear if the description could be more concise and focusing on how evolution sharped the metabolic flexibility. For example, the content of ‘It’s all about survival: Why evolution shaped metabolic flexibility’ seems discussed the interplay of metabolic programs in brain and other organs (e.g., skeletal muscle and adipocytes) which could be combined into the first section. The sections regarding uricase mutation could also be more straightforward by describing the importance of this mutation (e.g., more efficient metabolism of fructose and fat stores) in the way of evolution.  

**A: We have moved parts from the “Survival of the most flexible” section into the “It’s all about survival: Why evolution shaped metabolic flexibility’” section, and also joined both sections together to better connect the lines of reasoning provided in them. We have also re-structured and shortened the section on the uricase mutation in order to make our argumentation more comprehensible.**

 In the section of ‘Sedentary lifestyle’, the authors described a modern lifestyle which is opposite to the ‘The hunter and gatherer lifestyle’. Accordingly, this section could also benefit from a more concise storyline by comparisons such as sedentary behavior vs foraging mode.  Besides molecular-level evidence, there are also quite a lot of epidemiological evidence has highlighted the importance of environmental, and behavioral (dietary and sedentary behaviors) factors in the progress of metabolic health outcomes. Incorporation of these evidences will provide more insight into how to re-cultivate of suppressed evolutionarily metabolic programs and make the proposed model more convincing and readily understood for the researchers in the field as well as the general readers.

**A: We have removed some redundant text from the first subsection “Modern habitat: A life in mental stress and physical rest” in order to make the storyline and transition to the following subsections more concise. We have also added a total of five new references that provide additional confirmation of our hypothesis that reverting the sedentary lifestyle induces holistic beneficial metabolic effects.**

# Peer review: Referee response for version 1

**DOI:** 10.5256/f1000research.13782.r28024

**Published:** 2017-11-27

**Authors:** Johannes Coy

**Affiliations:** 1 Tavartis GmbH, Hainburg, Germany

The article ‘The sedentary (r)evolution: Have we lost our metabolic flexibility?’ by Freese and coworkers is an important contribution to solve the huge worldwide problem caused by a dramatic increase of diseases linked to nutrition and life style.

 The authors describe that during the course of evolution, up until the agricultural revolution, environmental fluctuations forced the human species to develop a flexible metabolism in order to adapt its energy needs to various climate, seasonal and vegetation conditions. During the longest time of *Homo sapiens*’ existence, humans’ daily survival was shaped by food insecurity (feast and famine), adaptation to a widespread range of different food sources (biodiversity), abundant daily exercise frequently under fasting conditions (foraging behavior), as well as unpredictable food supply (intermitted fasting), depending on the daily foraging success.

 The authors underline that the metabolic flexibility safeguarded human survival independent of changes in food availability and food composition. Furthermore the article shows the important and complex interplay and regulation of different metabolic pathways and a very important metabolic switch to the use of ketone bodies and fat oxidation.

 In the course of evolution, environmental stress forced the human species to develop an extraordinary flexible metabolism with multiple programs to guarantee energy equilibrium. Lipolysis, proteolysis, gluconeogenesis, ketone bodies and muscle-derived lactate as alternative energy substrates for cerebral neurons, provide examples by which natural selection tailored humans to buffer fluctuations in energy supply. Hormons as important key regulators of the metabolism as well as the role of the brain are being described in a very good and understandable way. In addition as a result of persistent psycho-emotional stressors the reaction of the brain concomitant with the changes in metabolism is shown. Furthermore the important impact of a lack of exercise is depicted.

 Due to this clear description of the metabolic flexibility and the absence of the use of this metabolic flexibility in modern humans following a Western life type, the authors hypothesize that the modern energetic inflexibility, typically displayed by symptoms of neuroglycopenia, can be reversed by re-activation of certain suppressed metabolic programs, which became obsolete in an affluent environment, particularly the ability to easily switch to ketone body and fat oxidation.

 The approach to re-activate metabolic programs represents an important and a not-well known approach to prevent and treat diseases related to metabolism and life style. Therefore this article is of utmost importance for scientists and clinicians in this field, but also enables individual humans to use the knowledge presented in this article to re-activate the metabolic programs by intermitting fasting, changes in food composition and/or exercises e.g. to activate the program to use ketone bodies and fat oxidation.

 There are a few terms which should be considered / could be changed to make the statements more clear.

**Abstract:**

 "In this article, we argue that the metabolic epidemic is largely based on a deficit in metabolic flexibility."

 The term ‘metabolic epidemic’ should be rephrased e.g.

 In this article, we argue that the strong increase of diseases related with an metabolic impact is largely based on a deficit in metabolic flexibility.

 The same should be done for the sentence in the introduction:

 "In this article, we argue that the metabolic epidemic in the developed world is based on a deficit in metabolic flexibility,…"

**Page 7**

 "Adipose tissue, responsible for 60% of all triacylglycerol accumulation, can store large amounts of excess FFA."

 The Abbreviation FFA is not being explained

**Page 9**

 "On a related note, changes in lifestyle patterns have been more successful in the context of a NAFL than pharmacological treatments, whereas exercise in isolation has not been proven to be effective."

 …the statement ‘whereas exercise in isolation has not been proven to be effective´ should be explained. The meaning of ‘exercise in isolation’ is not clear.

I have read this submission. I believe that I have an appropriate level of expertise to confirm that it is of an acceptable scientific standard.

# Response

**Published:** 2018-01-29

**Authors:** Jens Freese

**Affiliations:** Deutsche Sporthochschule Köln, Germany

**We would like to thank all reviewers for their constructive comments and positive feedback to our article. We have addressed their comments as described below, and think that they greatly helped to improve our article further.**

 "In this article, we argue that the metabolic epidemic is largely based on a deficit in metabolic flexibility."

 The term ‘metabolic epidemic’ should be rephrased e.g.

 In this article, we argue that the strong increase of diseases related with an metabolic impact is largely based on a deficit in metabolic flexibility.

 The same should be done for the sentence in the introduction:

 "In this article, we argue that the metabolic epidemic in the developed world is based on a deficit in metabolic flexibility,…"

**A: Done. We rephrased the term as “diseases related to metabolic abnormalities”**

**Page 7**

 "Adipose tissue, responsible for 60% of all triacylglycerol accumulation, can store large amounts of excess FFA."

 The Abbreviation FFA is not being explained

**A: We apologize and have replaced the abbreviation with “free fatty acids”**

**Page 9**

 "On a related note, changes in lifestyle patterns have been more successful in the context of a NAFL than pharmacological treatments, whereas exercise in isolation has not been proven to be effective."

 …the statement ‘whereas exercise in isolation has not been proven to be effective´ should be explained. The meaning of ‘exercise in isolation’ is not clear.

**A: The meaning should be „Exercise on its own without concurrent changes in diet”. However, the whole sentence was removed and replaced by a related sentence.**

# Peer review: Referee response for version 1

**DOI:** 10.5256/f1000research.13782.r27936

**Published:** 2017-11-27

**Authors:** Umesh Yadav, Johnna F. Varghese

**Affiliations:** 1 School of Life Sciences, Central University of Gujarat, Gandhinagar, India

The manuscript entitled “The sedentary (r)evolution: Have we lost our metabolic flexibility?” by Freese J. *et al.*, has discussed an important issue regarding the molecular and evolutionary reasons behind increased incidence of modern life style diseases in human beings. The authors have cited interesting studies to corroborate their points and delivered a convincing molecular and biochemical outlook for Western diseases (WD). The article makes an interesting read for the commoners as well as the scientific community.

 As far as the deficit in the metabolic flexibility in the modern times, as opposed to prehistoric times, is concerned the arguments given sound rational and justifiable, especially the switch between the fatty acid oxidation verses glucose oxidation in the hunter and gatherers of early times, which the authors argue has been lost in the modern times due to 1., availability of high glycemic food; 2., lack of extensive physical activities; 3., chronic psychological stress.

 The authors have discussed the relationship between the organs and shift in the metabolic flux during the two different lifestyles, hunter and gatherers, and sedentary. The metabolic linkages with circadian rhythm during hunter and gatherers life style, and how it has got disturbed now, are well explained.

 However, there are certain points authors may like to connect to make it more explicable. Take an example where the transition of life style was not abrupt, the hunter and gatherers when became agricultural societies and started dwelling in the stable communities with no uncertainty in most of the life activities, and feast and famine were not as frequent. Did the biochemical changes in the metabolism undergo some adaptation to the relatively and progressively ‘sedentary life style’? The agricultural societies have now transformed into industrialized societies with modern amenities and energy-rich foods within arm’s reach.

 Evolution indeed is a progressive natural mechanism. Is it possible that the ‘thrifty genes’ that became advantageous during hunting and gathering era of human existence, has become a sort of burden and will be eventually eliminated from the gene pool? Probably a better adapted gene set, say ‘lean genes’ to these emerging trends would help humans survive the endemic ‘modern life style’. Are there any indication of such evolutionary tendencies in humans today? Can the modern tools such as CRISPER-Cas be utilized in near future to modify the so called ‘thrifty genes’ and to rewire the metabolic flexibility? This is especially important when the evolution depends more on artificial selection than natural!

 The life, and the metabolism that drives life, are flexible and would therefore evolve an alternate mechanism to handle the pressure of modern life style on human health and survival. What biochemical alternatives or shunts could be suggested to overcome the so called metabolic inflexibility. Authors may have suggested or postulated more credible ways to thwart the metabolic inflexibility than merely propounding ‘retrain all prescribed modes periodically’, where the term ‘periodically’ is clearly vague!

 At places the authors have mentioned about the satiety signalling, where they state that leptin resistance is a common scenario in the sedentary life style. Is there evidence that the satiety signaling involving leptin and ghrelin have evolved in favour with the progressing inactive lifestyle?

 The authors have well explained the brain reward system in feeding behaviour. Modern habits and lifestyle is inclined towards abnormal hedonic behaviour where there is an excessive intake of processed food with high glycemic index and low nutritive value, dissatisfaction to the brain drives such a behaviour leading to obesity. Could authors suggest ways or quote studies where balancing the nutritive value of the food has been attempted such that they regulate the hormonal homeostasis that controls the excessive eating behaviour in individuals?

We have read this submission. We believe that we have an appropriate level of expertise to confirm that it is of an acceptable scientific standard.

# Response

**Published:** 2018-01-29

**Authors:** Jens Freese

**Affiliations:** Deutsche Sporthochschule Köln, Germany

**We would like to thank all reviewers for their constructive comments and positive feedback to our article. We have addressed their comments as described below, and think that they greatly helped to improve our article further.**

 The authors have discussed the relationship between the organs and shift in the metabolic flux during the two different lifestyles, hunter and gatherers, and sedentary. The metabolic linkages with circadian rhythm during hunter and gatherers life style, and how it has got disturbed now, are well explained.

 However, there are certain points authors may like to connect to make it more explicable. Take an example where the transition of life style was not abrupt, the hunter and gatherers when became agricultural societies and started dwelling in the stable communities with no uncertainty in most of the life activities, and feast and famine were not as frequent. Did the biochemical changes in the metabolism undergo some adaptation to the relatively and progressively ‘sedentary life style’? The agricultural societies have now transformed into industrialized societies with modern amenities and energy-rich foods within arm’s reach.

**A: Thank you for this comment. However, our point was not to imply that early agriculture led to better food security and less famine. In fact, early agriculturalists may have experienced more frequent and more severe periods of famine than hunter-gatherers due to the latter’s higher flexibility to change habitats (Berbesque et al. 2014, *Biol Lett* 10:** **20130853** **).  This also questions Neel’s thrifty gene hypothesis as we now briefly mention in the revised article. However, the lactase persistence gene and copy number variation in the *AMY1* (amylase) gene present examples for rapid biochemical adaptions that, however, only occurred in single genes and do not have a large impact on global physiology.**

 Evolution indeed is a progressive natural mechanism. Is it possible that the ‘thrifty genes’ that became advantageous during hunting and gathering era of human existence, has become a sort of burden and will be eventually eliminated from the gene pool? Probably a better adapted gene set, say ‘lean genes’ to these emerging trends would help humans survive the endemic ‘modern life style’. Are there any indication of such evolutionary tendencies in humans today? Can the modern tools such as CRISPER-Cas be utilized in near future to modify the so called ‘thrifty genes’ and to rewire the metabolic flexibility? This is especially important when the evolution depends more on artificial selection than natural!

**A: Even if there would be some true aspect to the thrifty gene hypothesis, we think it is very unlikely that evolution would now start to select for “lean genes”, for the following reasons: First, while obesity and its related diseases are now occurring more frequently at very young ages already, they are chronic in nature and do not pose a significant risk for dying before sexual reproduction has occurred. Second, modern medicines and pharmaceutical drugs are able to significantly prolong the life of patients with metabolic diseases. However, whether modern tools such as CRISPER-Cas could be utilized to modify certain “thrifty genes” remains speculative, and we think is beyond the scope of our paper. It would not only presuppose that such genes really exist (our example of uricase is rather unique in its effect on metabolism) but also that modifying these individual genes could reverse or neutralize the effects of an unhealthy lifestyle – which is probably not the case because lifestyle choices affect not individual genes, but have complex effects on the whole genome.**

 The life, and the metabolism that drives life, are flexible and would therefore evolve an alternate mechanism to handle the pressure of modern life style on human health and survival. What biochemical alternatives or shunts could be suggested to overcome the so called metabolic inflexibility. Authors may have suggested or postulated more credible ways to thwart the metabolic inflexibility than merely propounding ‘retrain all prescribed modes periodically’, where the term ‘periodically’ is clearly vague!

**A: We now mention pharmaceutical drugs such as metformin or “calorie restriction mimetics” such as resveratrol as possibilities that activate the starvation and foraging pathways (AMPK, SIRT1 etc.), but we think it is beyond the scope of this article to provide a somewhat detailed description of these approaches. Also, we are personally convinced that no single drug is able to fully mimic the complex interplay between molecular pathways that gets activated through lifestyle modifications. We have also removed the term “periodically” and replaced it with some more specific recommendation examples. ** 

 At places the authors have mentioned about the satiety signalling, where they state that leptin resistance is a common scenario in the sedentary life style. Is there evidence that the satiety signaling involving leptin and ghrelin have evolved in favour with the progressing inactive lifestyle?

 The authors have well explained the brain reward system in feeding behaviour. Modern habits and lifestyle is inclined towards abnormal hedonic behaviour where there is an excessive intake of processed food with high glycemic index and low nutritive value, dissatisfaction to the brain drives such a behaviour leading to obesity. Could authors suggest ways or quote studies where balancing the nutritive value of the food has been attempted such that they regulate the hormonal homeostasis that controls the excessive eating behaviour in individuals?
